# Emphysematous polycystic renal infection

**DOI:** 10.4103/0971-4065.73457

**Published:** 2010-10

**Authors:** Y. S. Sooraj, G. K. Nainan, F. Joseph, P. Thara

**Affiliations:** 1,2 Department of Nephrology, Lakeshore Hospital and Research Centre, Nettur PO, Kochi, India; 3 Department of Surgery, Co-operative Medical College and Hospital, Kochi, India; 4 Department of Radiology, Lakeshore Hospital and Research Centre, Kochi, India

**Keywords:** Autosomal dominant polycystic kidney disease, emphysematous polycystic renal infection, pyopneumocyst

## Abstract

Autosomal dominant polycystic kidney disease (ADPKD) is one of the commonest hereditary disorders. Urinary tract infection is a common complication of this disease. However emphysematous infections in ADPKD have seldom been reported. We report a case of emphysematous polycystic renal infection with Gram negative (*Escherichia coli*) septicemia in a nondiabetic patient with ADPKD who succumbed to his illness despite aggressive management including early nephrectomy.

## Introduction

Autosomal dominant polycystic kidney disease (ADPKD) is one of the most common hereditary disorders and it accounts for 8–10% of the cases of end-stage kidney disease (ESKD).[1] In this disease, the renal parenchyma is replaced by cysts of numerous sizes. Infection of the cyst is a common complication. However emphysematous infection of the cyst is a very rare entity and has seldom been reported.

## Case Report

**Figure 1:**
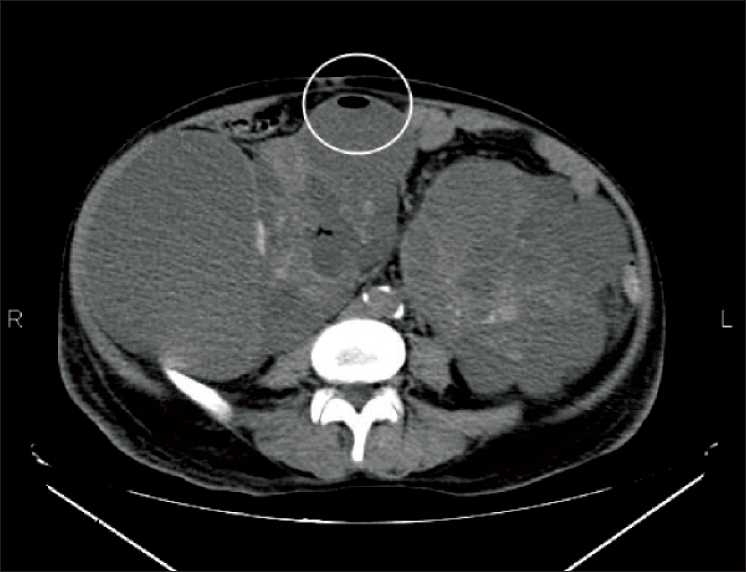
CT abdomen of the patient showing bilateral enlarged kidneys with multiple cysts. One of the cysts in the right kidney shows an air bubble (see circle)

A 57-year-old gentleman presented with history of high-grade fever with chills and rigors. He was recently detected to have ADPKD with hypertension and ESKD, and was on conservative management. He did not suffer from diabetes mellitus. Clinical evaluation revealed the presence of bilateral nodular abdominal masses. His pulse rate was 110/min and blood pressure was 100/70 mmHg. Investigations revealed a hemoglobin level of 4.7 g/dL, total leucocyte count of 27,000/cmm with 82% neutrophils. His blood urea was 230 mg/dL and serum creatinine was 8 mg/dL. Blood and urine cultures grew *Escherichia coli*. Ultrasonogram of the abdomen revealed grossly enlarged kidneys with multiple cysts. CT scan of the abdomen revealed bilateral polycystic kidneys with small calculi. One of the cysts in the lower pole of the right kidney showed an air bubble [Figure 1]. A diagnosis of Emphysematous polycystic renal infection (EPRI) was made. He was treated with higher antibiotics and underwent emergency nephrectomy. He also underwent continuous renal replacement therapy. Cutsection of the resected kidney revealed the presence of numerous cysts with serous hemorrhagic material and pus. The pus culture grew *E. coli*. However he did not recover and succumbed to his illness.

## Discussion

Approximately 30–50% of patients with ADPKD will have one or more renal infections during their life time.[2] However gas-forming infections in ADPKD is very rare. There have been only three case reports so far.[3 –5] *E. coli* and *Clostridium perfringens* have been demonstrated in different cases. The pus from the kidneys in our case grew *E. coli*.

Various theories have been proposed as to why gas forms inside cysts. In diabetics, CO_2_formation, resulting from the fermentation of the high concentration of sugar in the urine and tissue by infecting organisms, was regarded as the key factor of gas formation. However, the analysis of gas from infected cysts in a case of EPRI had shown only 4.1% CO_2_. Other composition was 10.5% oxygen, 67.3% nitrogen, and 18.1% unknown gas. This finding could not be fully explained by the sugar fermentation theory. Hence a new hypothesis was proposed. Impaired transportation of gas produced by rapid catabolism leads to gas accumulation in the tissue, which will gradually expand and create chambers to form gas bubbles. Gas of adjacent tissues will attempt to come into equilibrium with the gas bubbles. Positive equilibrium will lead to the continuous expansion of the lesion bubble. However, if the chamber is unable to withstand the increasing pressure then rupture or spontaneous drainage of the gas bubble may occur. During negative equilibrium, gas in the bubble gradually simulates tissue gas with eventual shrinkage of the bubble. If the chamber is unable to sustain the pressure, it collapses and the bubble disappears. However, if the chamber is capable of sustaining the pressure the bubble still may persist even when the gas content is equivalent to tissue gas. This may be the reason for pyopneumocysts in EPRI.[6]

EPRI should be differentiated from the emphysematous pyelonephritis (EPN) complicating ADPKD. EPN is a rare necrotizing form of acute renal infection that results in the presence of gas in the renal parenchyma, the perinephric tissue, or collecting system.[7] It is also more common in diabetics. EPN in ADPKD is also a very rare entity with only five cases reported so far.[8] The preexisting damaged renal tissue and impaired vascular supply act as a precipitating factor. The necrotic tissue is then used by certain bacteria as a substrate for gas formation. Hyperglycemia and impaired host response in sepsis contribute to it.[8] Urethral instrumentation was found to be the causative factor for bacterial seeding in one case.[9] The diagnosis is usually made by performing a CT scan of the kidneys, which denotes the presence of gas in the collecting system, perirenal tissue, and/or in the collecting system. In case of EPRI, gas will be present within the cysts. Conservative management with broad-spectrum antibiotics, adequate hydration, and circulatory support has shown to be beneficial especially in the cases picked up early and with less number of risk factors (thrombocytopenia, acute renal function impairment, disturbances of consciousness, and shock). However, percutaneous catheter drainage or surgical interventions including nephrectomy may be required in refractory cases or in the presence of obstruction.[8]

EPRI is one of the few indications for emergency nephrectomy in ADPKD. Antibiotic therapy alone may not be adequate because of the decreased antibiotic penetration into the cysts. But however, even with aggressive management, mortality rate remains high.

## Conclusion

Although urinary tract infections are common in ADPKD, EPRI is a very rare entity. To our knowledge, this is the fourth case being reported in literature. What makes this case even more unique is the fact that he is not a diabetic. Aggressive management including nephrectomy is warranted. However, prognosis remains grim despite all measures.
